# Spontaneous transomental hernia: a rare cause of closed loop bowel obstruction

**DOI:** 10.1093/jscr/rjaf034

**Published:** 2025-02-10

**Authors:** Caitlin Zhang, Marie Shella De Robles

**Affiliations:** Department of General Surgery, Shoalhaven District Memorial Hospital, Scenic Dr, Nowra, NSW 2541, Australia; Department of Surgery, The Wollongong Hospital, Loftus St, Wollongong, NSW 2500, Australia; Department of General Surgery, Shoalhaven District Memorial Hospital, Scenic Dr, Nowra, NSW 2541, Australia; University of Wollongong, Wollongong, Australia

**Keywords:** transomental hernia, closed loop bowel obstruction, internal hernia, diagnostic laparoscopy, spontaneous hernia

## Abstract

Transomental hernias are the rarest subtype of internal hernias, accounting for 0.5%–3% of bowel obstructions. We report an unusual case of a spontaneous transomental hernia in a 47-year-old male presenting with non-specific obstructive symptoms. A CT scan revealed a closed-loop small bowel obstruction, but the diagnosis of a spontaneous transomental hernia was confirmed during emergency diagnostic laparoscopy. The small bowel remained viable, avoiding the need for resection, and the patient had an uncomplicated postoperative recovery. Clinical suspicion for transomental hernias is crucial, especially in patients with no prior abdominal surgery, to ensure early surgical intervention and reduced morbidity.

## Introduction

Internal hernias are defined as the protrusion of viscera through a mesenteric or peritoneal aperture, a rare occurrence, accounting for only 0.5%–3% of bowel obstructions [9]. The most common subtypes include paraduodenal (53%) and pericaecal (13%), with less common occurrences involving the foramen of Winslow (8%), transmesenteric and transmesocolic (8%), intersigmoid (6%), and retroanastomotic (5%). Transomental hernias, where bowel protrudes through a defect in the omentum, are the rarest subtype, representing 1%–4% of internal hernias [6]. We present the case of a 47-year-old male with a closed-loop bowel obstruction secondary to a spontaneous transomental hernia.

## Case report

A 47-year-old Caucasian male presented to the Emergency Department with several hours of worsening upper abdominal pain. The pain was initially colicky but progressed to constant, with associated nausea but no vomiting or fever. He had a normal bowel motion the day prior to presentation. He had no comorbidities and no history of prior abdominal surgeries. All vital signs were within normal limits, and he was afebrile. On examination, his abdomen was mildly distended, soft, and tender to palpation on the left side and centrally. His white cell count was elevated at 13.2 × 10^9^/L, with normal C-reactive protein, liver function tests, lipase, pH, and lactate of 1.2.

**Figure 1:**
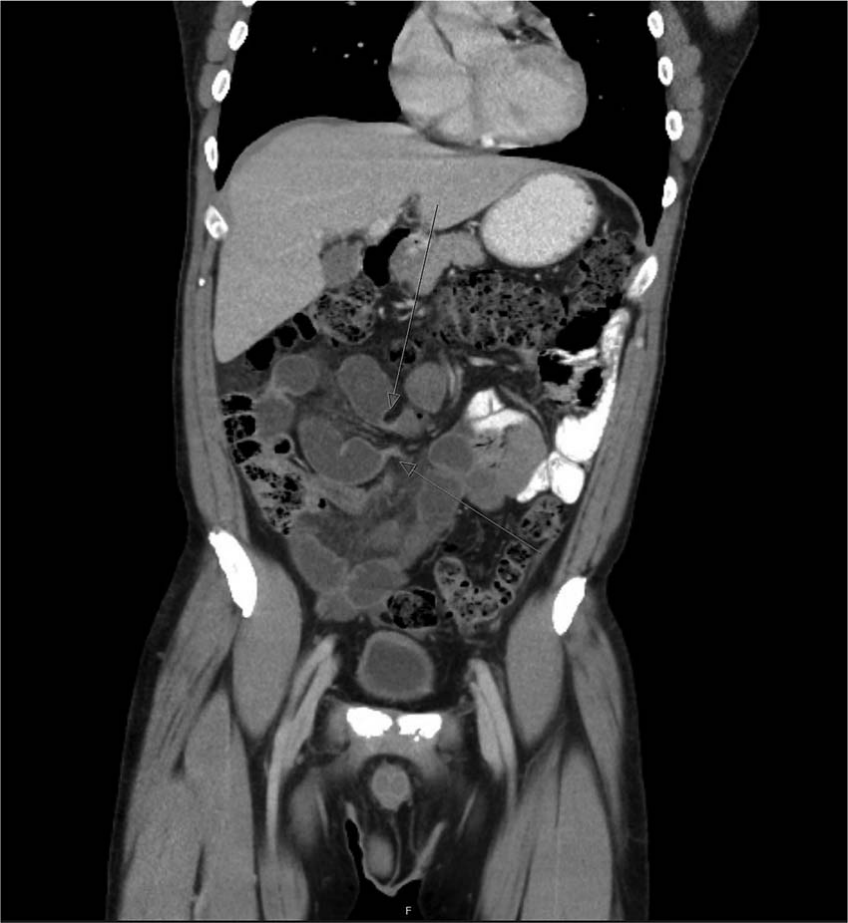
Abdominal CT scan demonstrating closed-loop small bowel obstruction involving jejunal loops, with two distinct transition points indicated by arrows.

A CT abdomen and pelvis with oral and intravenous contrast revealed a closed-loop small bowel obstruction, with dilated loops of small bowel in the right mid to lower abdomen and associated mesenteric fluid (Fig. 1). A nasogastric tube was inserted, and the patient was taken for emergency surgery.

**Figure 2:**
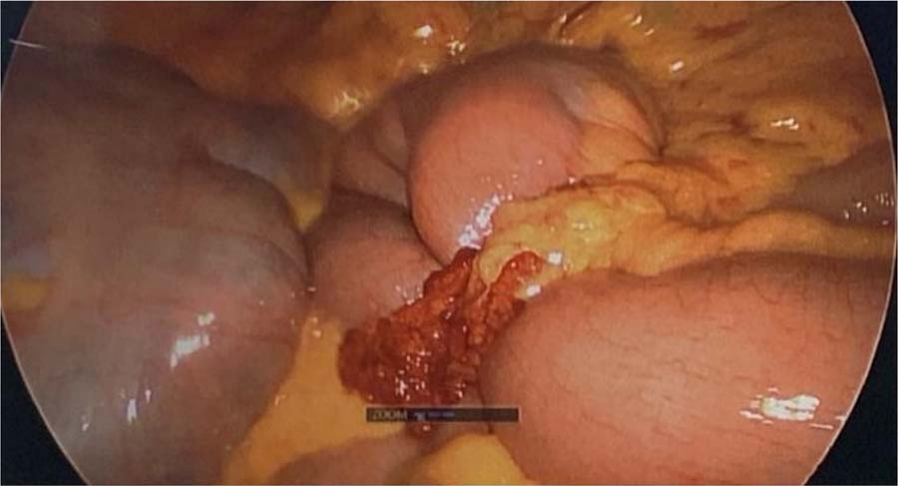
Intraoperative image from diagnostic laparoscopy showing a loop of jejunum herniating through a defect in the greater omentum.

**Figure 3:**
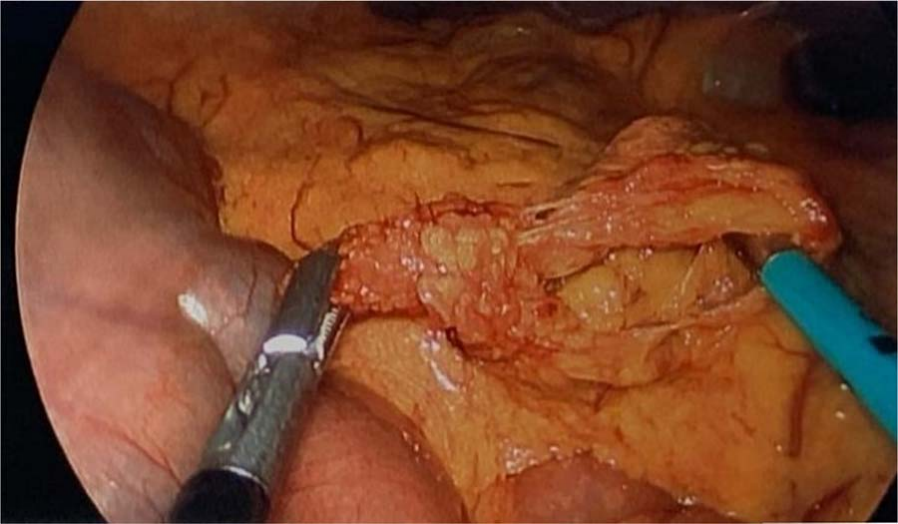
Intraoperative image showing the omental defect after reduction of the herniated loop of jejunum.

During diagnostic laparoscopy, a loop of jejunum was found to be transomentally herniated, congested but viable (Figs 2 and 3). Adhesiolysis was performed, and no small bowel resection was required. The patient was discharged on postoperative day 2 after an uneventful recovery and has not experienced any complications since.

## Discussion

Transomental hernias are a rare cause of small bowel obstruction, with only 24 cases reported in the English literature over 60 years [5]. They tend to present in a bimodal age distribution, affecting pediatric patients and adults over 50 years of age. Omental defects may be congenital, due to senile atrophy, or acquired from surgery, trauma, or inflammation [9, 12]. Yamaguchi classified transomental hernias into three types: A (through the fused layers of the greater omentum), B (through the omental bursa), and C (into the omental bursa) [11].

Diagnosing transomental hernias preoperatively is difficult [9], as symptoms mimic other intestinal obstructions—abdominal pain, nausea, vomiting, distension, and obstipation [5, 6]. However, they have a higher risk of strangulation due to the small orifice, leading to up to 30% morbidity [8]. CT is the most common imaging modality, often showing dilated bowel loops, collapsed distal bowel segments, and the ‘beak sign,’ indicative of a triangular-shaped transition zone [2]. Other findings include the ‘whirl sign,’ representing a swirling pattern of mesenteric vessels [1]. Localization of dilated small bowel loops in the lesser sac suggests transomental hernia, though definitive diagnosis often requires surgery [4, 6].

Surgical reduction of the herniated bowel is the treatment of choice, performed either laparoscopically or via laparotomy [7]. Laparoscopy is typically possible, given that proximal jejunal loops are usually involved, providing adequate space for pneumoperitoneum [4, 5, 10]. Small bowel resection is required if ischemia or necrosis is present. Repair of the omental defect, often with sutures or partial omentectomy, helps prevent recurrence [3].

## Conclusion

Transomental hernias are a rare cause of small bowel obstruction, often presenting with nonspecific clinical and radiological signs. Maintaining a high index of suspicion is essential for early surgical intervention and reducing morbidity.
